# Combined vaccine-immune-checkpoint inhibition constitutes a promising strategy for treatment of dMMR tumors

**DOI:** 10.1007/s00262-021-02933-4

**Published:** 2021-04-18

**Authors:** Inken Salewski, Steffen Kuntoff, Andreas Kuemmel, Rico Feldtmann, Stephan B. Felix, Larissa Henze, Christian Junghanss, Claudia Maletzki

**Affiliations:** 1 Department of Medicine Clinic III - Hematology, Oncology, Palliative Medicine, Rostock University Medical Center, Ernst-Heydemann-Str. 6, 18057 Rostock, Germany; 2 Department of Internal Medicine B, Cardiology, University Medicine Greifswald, Greifswald, Germany; 3 DZHK (German Centre for Cardiovascular Research), Partner Site Greifswald, Greifswald, Germany

**Keywords:** *α*-PD-L1, MMR deficiency, In vivo imaging, Tumor microenvironment, Long-term survival

## Abstract

**Background:**

Mlh1-knock-out-driven mismatch-repair-deficient (dMMR) tumors can be targeted immunologically. By applying therapeutic tumor vaccination, tumor growth is delayed but escape mechanisms evolve, including upregulation of immune-checkpoint molecules (LAG-3, PD-L1). To counteract immune escape, we investigated the therapeutic activity of a combined tumor vaccine-immune-checkpoint inhibitor therapy using *α*-PD-L1.

**Design:**

In this trial, Mlh1-knock-out mice with established gastrointestinal tumors received single or thrice injections of *α*-PD-L1 monoclonal antibody clone 6E11 (2.5 mg/kg bw, q2w, i.v.) either alone or in combination with the vaccine. Longitudinal flow cytometry and PET/CT imaging studies were followed by ex vivo functional immunological and gene expression assays.

**Results:**

6E11 monotherapy slightly increased median overall survival (mOS: 6.0 weeks vs. control 4.0 weeks). Increasing the number of injections (*n* = 3) improved therapy outcome (mOS: 9.2 weeks) and was significantly boosted by combining 6E11 with the vaccine (mOS: 19.4 weeks vs. 10.2 weeks vaccine monotherapy). Accompanying PET/CT imaging confirmed treatment-induced tumor growth control, with the strongest inhibition in the combination group. Three mice (30%) achieved a complete remission and showed long-term survival. Decreased levels of circulating splenic and intratumoral myeloid-derived suppressor cells (MDSC) and decreased numbers of immune-checkpoint-expressing splenic T cells (LAG-3, CTLA-4) accompanied therapeutic effects. Gene expression and protein analysis of residual tumors revealed downregulation of PI3K/Akt/Wnt-and TGF-signaling, leading to T cell infiltration, reduced numbers of macrophages, neutrophils and MDSC.

**Conclusions:**

By successful uncoupling of the PD-1/PD-L1 axis, we provide further evidence for the safe and successful application of immunotherapies to combat dMMR-driven malignancies that warrants further investigation.

**Supplementary Information:**

The online version contains supplementary material available at 10.1007/s00262-021-02933-4.

## Background

Immunotherapy with immune-checkpoint inhibitors (ICI) has become a mainstay of treatment for a range of solid cancers, including melanoma, bladder cancer, non-small cell lung cancer and Hodgkin’s lymphoma [1–5]. CTLA-4, PD-1, or PD-L1 are the so far most studied checkpoint molecules and ICI widely applied in the clinic to improve patients’ prognosis. This blockade reactivates exhausted T-cells, prevents T-cell inhibition, and promotes effector T-cell proliferation to stimulate T-cell-mediated tumor cell killing [6–8]. Atezolizumab, Avelumab and Durvalumab are FDA approved as PD-L1 blocking antibodies. Monotherapy results in antitumor immune responses yet have a limited long-term therapeutic efficacy in most cases.

Lessons learned from the last years identified mismatch-repair deficiency (dMMR) as a molecular subtype with high response rates toward ICI. DMMR-driven carcinogenesis emerges sporadically because of MMR gene promoter hypermethylation or as part of defined hereditary tumor syndromes such as Lynch Syndrome and constitutional mismatch-repair deficiency [9–14]. The spectrum of cancer types related to dMMR is complex and includes, among others, gastrointestinal, endometrial and urothelial cancers [15]. A hallmark of dMMR tumors – irrespective of organ manifestation – is an ultramutated tumor phenotype (= TMB high), leading to a high abundance of frameshifted neo-epitopes on the tumor cells’ surface. This latter feature underlines the tremendous potential for immunological targeting of dMMR cancers [15–17]. Indeed, in 2017, the FDA approved *α*-PD-1 ICI Pembrolizumab and Nivolumab for treatment of dMMR cancers agnostic of cancer site [18], which was extended lately for the first-line treatment of patients with un-resectable or metastatic dMMR colorectal cancer (CRC). Pre-existing Th type1 immune responses and high numbers of tumor-infiltrating CD8^+^ T-cell clones (= IFN*γ* signature) constitute positive predictive biomarkers [19]. However, roughly 25% of patients show intrinsic resistance and in most cases initially responding patients gradually develop resistance, highlighting the necessity of improving treatment options [20–23]. As for PD-L1, limited preclinical data exist. PD-L1 expression on tumor-infiltrating lymphocytes is thought to be a potential predictor for patients’ response to *α*-PD-1 therapy, but it is not well established for dMMR cancers because of the generally low expression [24, 25]. A recent phase II study in patients with dMMR metastatic or unresectable CRC revealed antitumor activity of Avelumab monotherapy [26]. Additional clinical trials are ongoing with different combinations being employed. One of them is based on tumor lysates or specific neoantigen-derived peptides. The former act as “global” vaccines and induce objective responses in some patients. To refine combination approaches preclinically, we employed the Mlh1 knock-out mouse model for dMMR-related diseases. Preceding vaccination approaches yielded prolonged overall survival in the therapeutic and prophylactic setting [27, 28]. Residual tumor cells showed an upregulation of immune-checkpoint molecules as part of acquired resistance. To counteract vaccination-induced immune escape and improve overall survival, we here applied a murine *α*-PD-L1 antibody (clone: 6E11) in combination with repeated vaccination.

## Methods

### Cell culture & vaccine preparation

Cells were cultured in DMEM medium, supplemented with 10% FCS (fetal calf serum), 6 mM Glutamine, and antibiotics (all from Biochrom, Berlin, Germany). The tumor lysate was prepared from a A7450 tumor allograft as described [29].

### Mlh1^−/−^ mouse model and in vivo treatment protocol

#### Ethical statement

The German local authority approved all animal experiments: Landesamt für Landwirtschaft, Lebensmittelsicherheit und Fischerei Mecklenburg‐Vorpommern (7221.3‐1‐026/17; -026/17‐3), under the German animal protection law and the EU Guideline 2010/63/EU. Mice were bred in the animal facility of the University Medical Center in Rostock under specific pathogen‐free conditions. Mlh1 genotyping was done according to [21]. During their whole lifetime, all animals got enrichment in the form of mouse-igloos (ANT Tierhaltungsbedarf, Buxtehude, Germany), nesting material (shredded tissue paper, Verbandmittel GmbH, Frankenberg, Germany), paper roles (75 × 38 mm, H 0528–151, ssniff‐Spezialdiäten GmbH), and wooden sticks (40 × 16 × 10 mm, Abedd, Vienna, Austria). During the experiment, mice were kept in type III cages (Zoonlab GmbH, Castrop‐Rauxel, Germany) at 12‐h dark:light cycle, the temperature of 21 ± 2 °C, and relative humidity of 60 ± 20% with food (pellets, 10 mm, ssniff‐Spezialdiäten GmbH, Soest, Germany) and tap water ad libitum.

### Experimental protocol

Mice with PET/CT proven gastrointestinal tumors (GIT), located in the duodenum, were conducted to therapy using four weekly tumor lysate boosts. Vaccination was sustained (10 mg/kg bw, biweekly, *n* = 10 mice) until tumors progressed, but for a maximum of 12 times. Treatment with *α*-PD-L1 (clone 6E11, kindly provided by Genentech, a subsidiary of Roche, South San Francisco, USA, dissolved in PBS) given at 2.5 mg/kg bw intravenously was done once (*n* = 4 mice) or thrice (*n* = 10 mice) every second week (q2wx3). Mice receiving the combination of *α*-PD-L1 were given vaccine first, followed by *α*-PD-L1 injection. Here again, combinations included single or triple *α*-PD-L1 applications (*n* = 10 mice/group; q2wx1 and q2wx3). Control mice were left untreated (*n* = 10 mice). Reduction of suffering was guaranteed by providing daily prepared soaked pellets, twice-daily monitoring of the health status using a score sheet and by applying humane endpoints (weight loss > 15%, pain/distress, changes in social behavior). All mice were sacrificed before they became moribund to prevent pain and distress. At this time, blood samples, spleens, lymph nodes and GIT were removed for further analyses.

### PET/CT imaging

PET/CT imaging scans were performed on a small animal PET/CT scanner (Inveon PET/CT, Siemens Medical Solutions, Knoxville, TN, USA) according to a standard protocol as described before [30]. Briefly, mice were anesthetized by isoflurane (1–3%, supplemented with oxygen) and received a mean dose of 16.03 ± 1.10 MBq ^[18F]^FDG intravenously via a microcatheter placed in a tail vein. Static PET scans were acquired using a small animal micro PET/CT scanner (Inveon PET/CT Siemens, Knoxville, TN, USA). The PET image reconstruction method consisted of a 2-dimensional ordered subset expectation maximization algorithm (2D-OSEM) with four iterations and six subsets. Attenuation correction was performed on the basis whole body CT scan and a decay correction for [^18^F] was applied. PET images were corrected for random coincidences, dead time and scatter. By marking the entire tumors, starting at the edge and cutting through the whole ^[18F]^FDG-enriched tumor, volumes and SUVs were determined. This was done by using Inveon Research Workplace 4.2 software.

### Immune phenotyping

Blood samples were taken routinely from the retrobulbar venous plexus. Single cell suspensions of spleens and GIT were obtained upon passing them through a cell strainer (100 µm). Samples (2 × 10^5^/Well) were stained with a panel of conjugated monoclonal antibodies (mAb, 1 μg each) followed by lysis of erythrocytes (155 mM NH_4_Cl (MERCK Millipore, Darmstadt, Germany), 10 mM KHCO_3_ (MERCK Millipore) and 0.1 mM EDTA (Applichem, Darmstadt, Germany). Negative controls consisted of lymphocytes stained with the appropriate isotypes (Biolegend, San Diego, USA). Cells were washed, resuspended in PBS and analyzed by flow cytometry on a Flow Cytometer (BD FACSVerse™, BD Pharmingen). Data analysis was performed using BD FACSuite software (BD Pharmingen).

### Procartaplex cytokine assay

Cytokine levels in plasma samples were determined according to the manufacturer’s instructions of the Procartaplex™ multiplex immunoassay (Thermo Fisher Scientific, Schwerte, Germany). Measurement as well as cytokine quantification was performed on a Bioplex 2000 (Bio-Rad Laboratories GmbH, Munich, Germany) in combination with the BioPlex Manager Software. Absolute plasma cytokine and chemokine level are presented [ng/ml].

### Fragment length analysis of cMS target genes

A panel of non-coding and coding MS marker was analyzed as described before [31]. MSI is defined by mono- and/or bialellic band shifts usually characterized by deletions (indicated with minus symbol + number).

### Nanostring targeted gene expression profiling

The T cell–inflamed tumor microenvironment was analyzed by targeted gene expression profiling of tumor RNA from fresh frozen or Tissue-Tek® embedded treatment and control samples (*n* = 3 samples/group). Total RNA was isolated using the RNeasy Mini Kit according to the manufacturers’ instruction (Qiagen, Hilden, Germany). Total RNA concentrations were measured using the NanoDrop ND1000 (Thermo Fisher Scientific). Gene expression analysis was conducted on the NanoString nCounter gene expression platform (NanoString Technologies, Seattle, WA) applying the PanCancer IO 360™ Panel. This panel enables digital profiling of 770 genes that shape the tumor-immune interface and allows for characterization of pathways relevant in immune response and escape. Quality control, normalization and data analysis was done by applying the nSolver™ Analysis Software 4.0 including nCounter Advanced Analysis (version 2.0.115). Data are presented as Heatmap and log10 (*p *value) as well as log2 fold change.

### Immunofluorescence

Cryostat sections of 4 μm were air-dried and fixed in cold pure methanol for 8 min. Unspecific binding sites were blocked in 2% BSA (Roth) for 2 h followed by incubation with 1 μg of the following FITC- and PE-labeled mAbs: CD4, CD8α, CD11b, Gr1 (Immunotools, Friesoythe, Germany), CD11c, CD104, LAG-3, PD-1, F4/80 and PD-L1 (Biolegend). Sections were washed, embedded in Roti Mount Flour Care DAPI (Roth, Karlsruhe)and target proteins visualized on a confocal laser scanning microscope (LSM780, Zeiss, Jena, Germany) using 20× objectives.

### IFN-*γ* ELISpot

2.5 × 10^3^ targets/well (2 GIT cell lines: Mlh1^−/−^ A7450, Mlh1^−/−^ 328, 1 lymphoma cell line: Mlh1^−/−^ 1351, and YAC-1 cells) were seeded in IFNγ–specific mAb (Mabtech, 3321–3)–coated, 96-well microtiter plates. Peripheral blood leukocytes (5 × 10^4^/Well) or splenocytes (1 × 10^4^/well) from vaccinated and control mice were added in triplicates and co-cultured overnight. Bound antibody (Mabtech, 3321–6) was visualized by BCIP/NBT (KPL, Gaithersburg, Maryland, USA); spots were counted using an ELISpot reader. Presented are the numbers of IFNγ–secreting cells per 10,000 effector cells corrected for background levels counted in the absence of target cells, which was always ≤ 5 spots/well. Target cells without effector cells showed no background level.

### Statistics

All values are expressed as mean ± SD. After proving the assumption of normality (Kolmogorov–Smirnov test), differences between vaccinated and control mice were determined using the unpaired Student’s t test or one-way ANOVA (Bonferroni or Dunnett’s multiple comparison). Kaplan–Meier survival analysis was done by applying the log rank (Mantel Cox) test. Statistical analyses were performed using GraphPad Prism 5 (San Diego, CA). The criterion for significance was set to *p* < 0.05.

## Results

### Combination of *α*-PD-L1 and vaccines significantly improves outcome of Mlh1^−/−^ mice

**Fig. 1:**
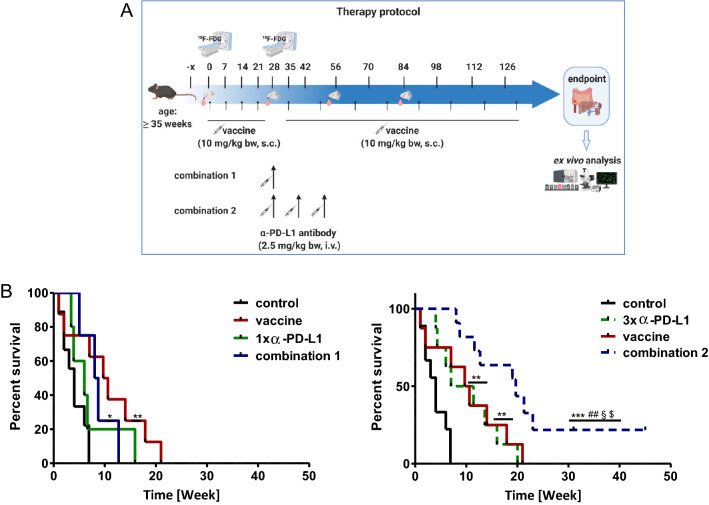
Therapy protocol and Kaplan–Meier survival curve **a** Schematic overview on the treatment protocol, including time points for blood collection and PET/CT imaging **b** Log rank survival analysis of treated and control mice. Mice with confirmed GIT received four weekly injections of the tumor lysates (= vaccine; 10 mg/kg bw, biweekly, *n* = 10 mice) until tumors progressed (maximum 12 injections). Treatment with *α*-PD-L1 (clone 6E11, Genentech) given at 2.5 mg/kg bw intravenously was done **a** once (*n *= 4 mice) or **b** thrice (*n* = 10 mice) every second week (q2wx3). Mice receiving the combination were given vaccine first, followed by *α*-PD-L1 injection. Here again, combinations included **a** single or **b** triple *α*-PD-L1 applications (*n* = 10 mice/group). Control mice were left untreated (*n* = 10 mice). *p < 0.05 versus control; **p < 0.01 versus control; ***p < 0.001 versus control; ^##^*p* < 0.01 versus 1x *α*-PD-L1; §*p* < 0.05 versus 3x *α*-PD-L1; $*p* < 0.05 versus vaccine

In a first cohort, the ICI *α*-PD-L1 was administered once because of its long half-value period. Effects on survival were only marginal (Fig. 1a) and may indicate that MLH1^−/−^- associated tumors are refractory to ICI monotherapy. By combining single *α*-PD-L1 with the vaccine (combination 1), overall survival was not significantly improved. In the next step, *α*-PD-L1 was given three times, to see whether tumors are indeed ICI-refractory or single application was simply not sufficient to induce immune responses in this model. Indeed, thrice *α*-PD-L1 injection extended the life span of mice to a degree comparable to the vaccine monotherapy (Fig. 1b). In combination with the vaccine (= combination 2), *α*-PD-L1 antibody treatment could even quintuple the life of mice from four weeks (control) to ~ 20 weeks (*p* < 0.001 vs. control; *p* < 0.01 vs. 1x *α*-PD-L1; *p* < 0.05 vs. 3x *α*-PD-L1; *p* < 0.05 vs. vaccine). Hence, this combination partially abrogated intrinsic ICI resistance and we consequently continued to move on with the triple *α*-PD-L1 treatment (combination 2, hereafter referred as combination) for subsequent functional analyses.

### Combinational therapy leads to tumor reduction

**Fig. 2:**
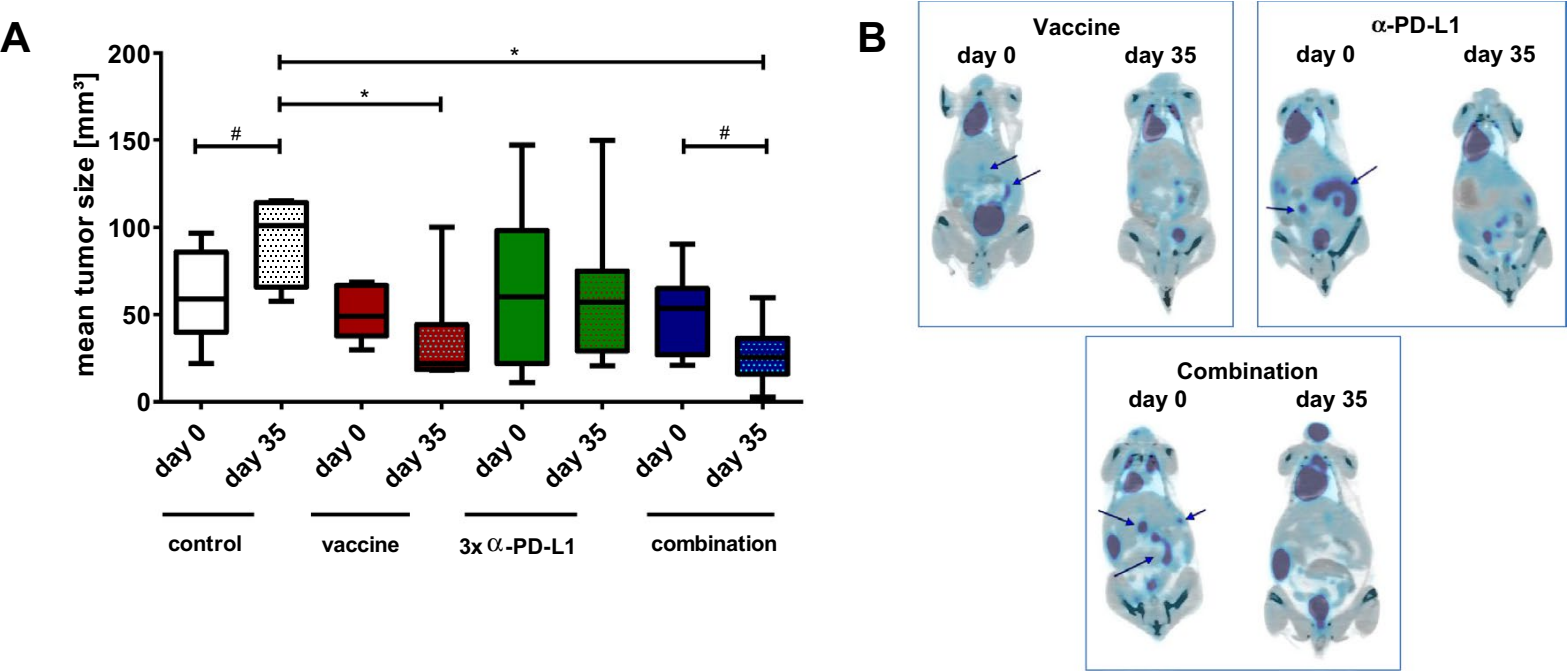
PET/CT imaging **a** PET/CT analysis for quantification of mean tumor volume [mm^3^]. Represented are the mean tumor sizes ± SD at start of treatment and after 28 days of treatment (*n* = 4–10 mice/group and time-point); *p < 0.05 versus control, one-way ANOVA (Bonferroni’s multiple comparison test); #*p* < 0.05 versus day 0; t-test **b** Representative PET/CT scans from mice receiving the vaccine (upper left), the a-PD-L1 antibody (upper right) or a combination of both (lower). Arrows indicate measurable tumor nodules in the gut

Longitudinal PET/CT measurement revealed significant tumor size reduction by either therapy (vaccine, *α*-PD-L1 and combination) compared to controls (Fig. 2a). Still, analysis of the tumor size within the treatment groups identified significant reduction over time only in the combination (Exemplary pictures are given in Fig. 2b) finally resulting in partial or even complete remission. For the latter, this promising result was seen in three mice. Two of them remained alive until the experimental endpoint and one mouse had to be euthanized because of a progressive cutaneous benign lesion (week 23).

### Peripheral immune activation by vaccine-based immunotherapy

**Fig. 3:**
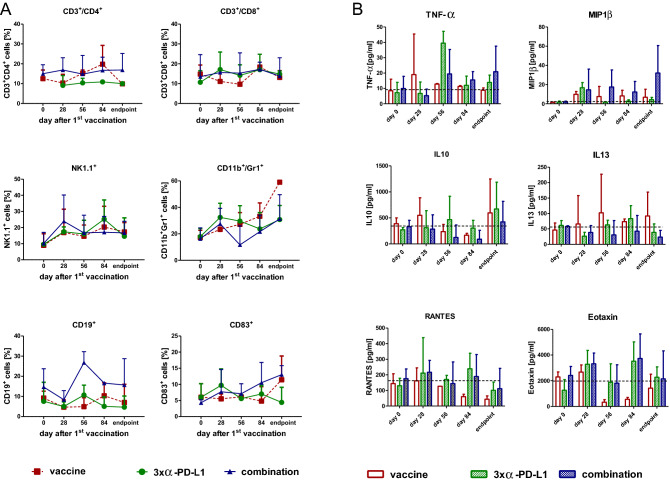
Longitudinal flow cytometric phenotyping and plasma cytokine level **a** Phenotyping of peripheral blood leukocytes was done before, during therapy and at the endpoint. Therefore, blood was collected via the retrobulbar venous plexus and stained with antibodies. Given are the percentage numbers of positive cells ± SD resulting from 20,000 events measured on a flow cytometer. **b** Plasma cytokine levels from mice with immunotherapy and controls (upper graph). Differences between tumor-free and tumor-bearing mice (lower graphs). Plasma samples were collected at the experimental endpoint and cytokine levels determined as described in material and methods. Given are the mean cytokine level ± SD

To investigate the immunological changes during therapy, blood was taken from mice every four weeks and analyzed via flow cytometry (Fig. 3a). The vaccine treatment-induced a temporary increase in CD3^+^/CD4^+^ T-helper cells at day 84 which was not seen in the other groups. The level of CD3^+^/CD8^+^ cytotoxic T-lymphocytes (CTL) remained constant over time, while the amount of NK cells increased continually in all three treatment groups. The CD11b^+^/Gr1^+^ MDSC was doubled during the therapy with every treatment. The effects on CD19^+^ B-lymphocytes were oppositional. B-lymphocytes increased in the combination and decreased during vaccine or *α*-PD-L1 treatment. CD83^+^ dendritic cells (DC) were mainly found in mice treated with the vaccine only or the combination, likely because of stimulating the humoral arm of the immune system.

To investigate changes in the cytokine levels that act as growth factors, we analyzed plasma levels from different time points and at the end using a multiplex cytokine assay. TNF-*α* showed only marginal changes with the vaccine, a remarkable peak at day 56 in the *α*-PD-L1 treatment and a constant slight increase over time in the combination (Fig. 3b). This latter increase was also seen for the chemoattractant MIP1β. The IL10 level fluctuated in all three treatments. In contrast, the vaccine-induced IL13, while it remained unaffected upon *α*-PD-L1 treatment and decreased in the combination group, indicative for minor relevance of Th2-cytokines in treatment response. The levels of RANTES and Eotaxin decreased with vaccination, but for *α*-PD-L1 and the combination it initially increased.

### Changes in important sites for immune reactions: spleens and residual tumors

**Fig. 4:**
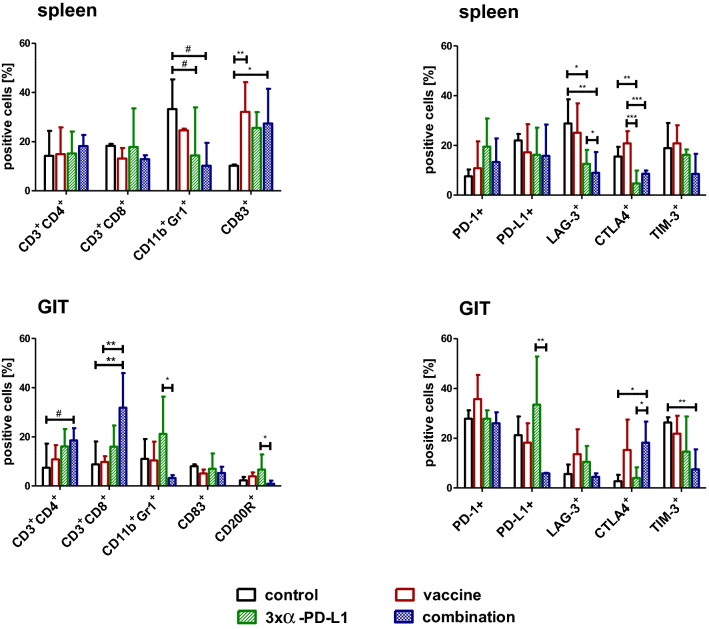
Flow cytometric phenotyping of spleen and GIT cells. Phenotyping was done at the endpoint. Therefore, mice were euthanatized, organs removed and single cell suspensions stained with the appropriate antibodies. Given are the percentage numbers of positive cells ± SD resulting from 20,000 events measured on a flow cytometer. *p < 0.05; **p < 0.01; ***p < 0.001 one-way ANOVA (Bonferroni’s multiple comparison test); ^#^*p* < 0.05 one-way ANOVA (Dunnett’s multiple comparison test)

Additionally to the blood immune-monitoring, spleens and residual tumors were resected at the experimental endpoint and infiltrating cells analyzed by flow cytometry. In spleens, levels of CD3^+^/CD4^+^ T-helper cells and CD3^+^/CD8^+^ CTL did not change with the therapies. By contrast, levels of CD11b^+^/Gr1^+^ MDSC decreased and the CD83^+^ DC significantly increased in all treatment groups with a trend toward stronger effects in the combination (*p* < 0.05; and *p* < 0.01 vs. control; Fig. 4). The amount of PD-1^+^ cells increased slightly, PD-L1^+^ cells reduced. LAG-3^+^ cells decreased significantly, especially in the *α*-PD-L1 and the combination treatment (*p* < 0.05; and *p* < 0.01 vs. control). The same effect was seen for CTLA4^+^ cells (*p* < 0.05; *p* < 0.01; *p* < 0.001 vs. control and vaccine) and TIM-3^+^ cells.

Residual tumors harbored higher numbers of infiltrating CD3^+^/CD4^+^ T-helper cells and CD3^+^/CD8^+^ CTL, particularly for the combination group. CD11b^+^/Gr1^+^ MDSC increased with *α*-PD-L1 treatment and dropped in the combination (*p* < 0.05 vs. *α*-PD-L1). Levels of CD83^+^ DC were constant and similar to the control, while CD200R^+^ cells reduced in the combination (*p* < 0.05 vs. *α*-PD-L1). Looking at the frequency of immune-checkpoint molecules, there were additional differences. The abundance of PD-1^+^ cells remained unchanged in all groups, PD-L1^+^ cells increased with *α*-PD-L1 treatment and decreased in the combination (*p* < 0.01 vs. *α*-PD-L1). Infiltrating LAG-3^+^ cell numbers were high in the monotherapies, whereas CTLA4^+^ infiltration was mainly confined to groups of the vaccine (vaccine monotherapy and combination). Still, TIM-3^+^ cells decreased significantly upon combination (*p* < 0.01 vs. control).

### Gene expression analysis identifies downregulation of PI3K/Akt/Wnt-and TGF-signaling

**Fig. 5:**
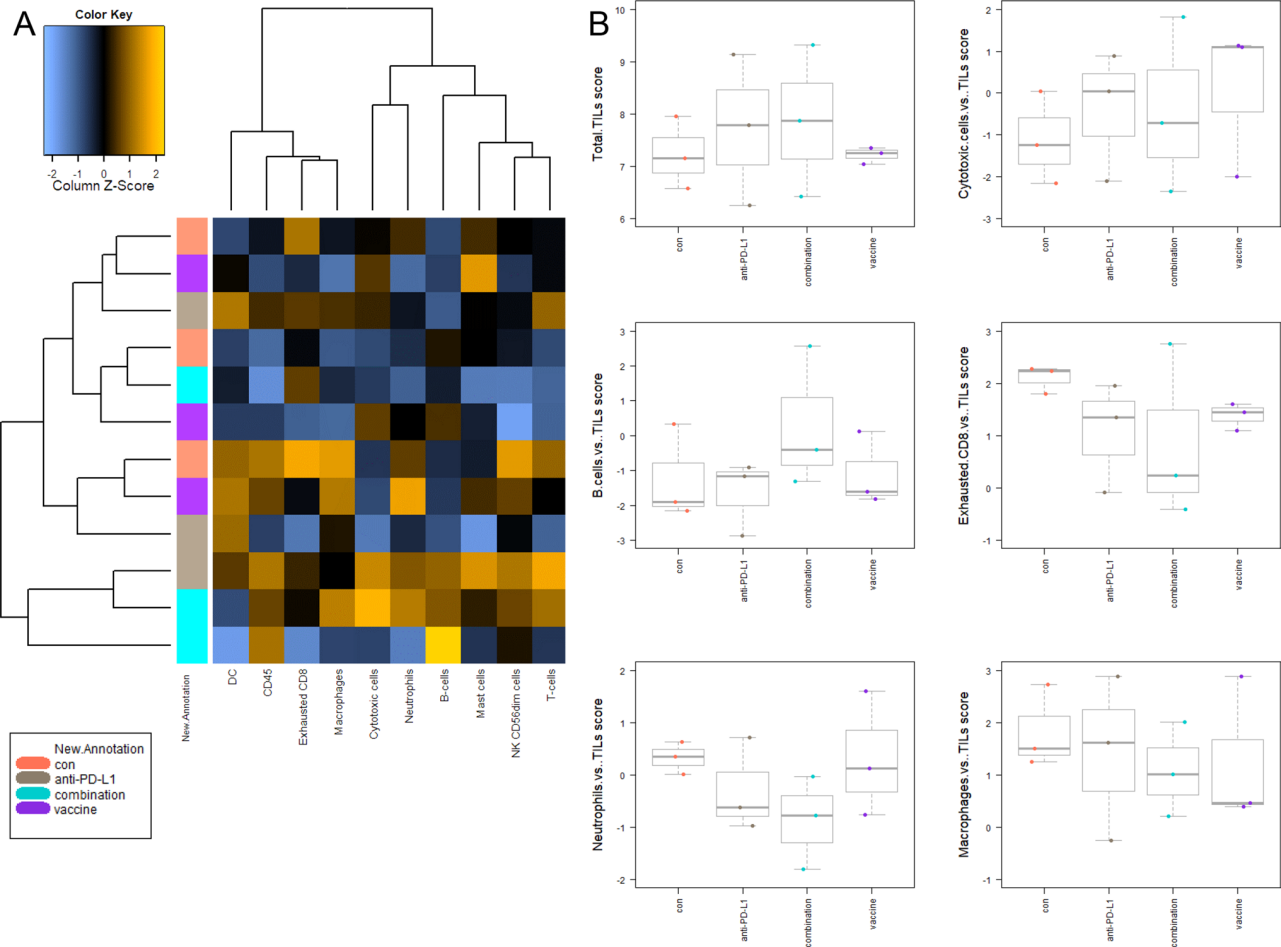

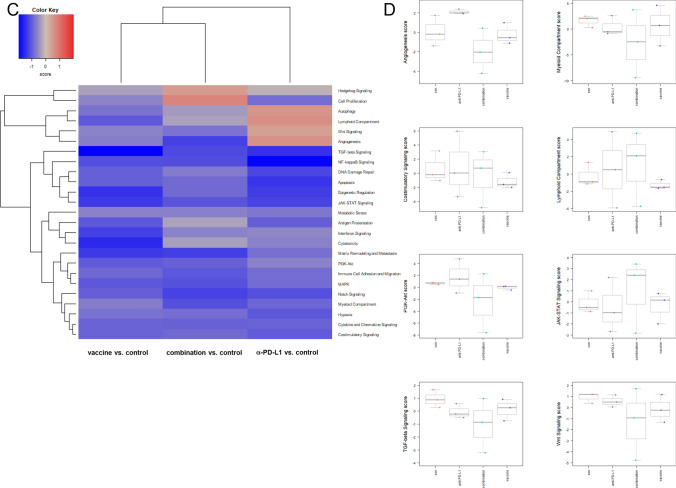
PanCancer gene expression analysis of treated and control tumors **a** Heatmap showing raw abundance of cell types in each sample. Orange shows high abundance; blue indicates low abundance **b** Relative abundances measuring various contrasts between cell types reported for each group. Data result from *n* = 3 samples/group. (**c, d**) Affected pathways in treated and control tumors **c** Directed global significance statistics measure the extent to which a gene set's genes are up or downregulated with the variable. Red denotes gene sets whose genes exhibit extensive over-expression with the covariate, blue denotes gene sets with extensive under-expression **d** Scores of selected pathways shown for each group. Increasing scores correspond to an increasing expression. Data result from *n* = 3 samples/group

To have a closer look on the complex interplay between the tumor microenvironment and immune response, the PanCancer IO 360 Gene Expression Panel was applied (Fig. 5).

The cluster, left to the heat map (Fig. 5a), schematically illustrates the relations of the three individuals of the four treatment groups dependent on their tumor-infiltrating lymphocyte (TIL) levels. The amounts of different immune cells differ the most in the combination group compared to the other groups. Here, overall immune cell expression levels increased in two of three individuals. Treatment with *α*-PD-L1 also changed immune cell expression patterns compared to the control and vaccine treatment.

Total TIL levels (Fig. 5b, upper left) were elevated in the *α*-PD-L1 and the combination therapy, because of increasing amounts of cytotoxic T and B cells (upper and middle). Conspicuously, levels of exhausted CD8^+^ T cells and neutrophils exclusively decreased in the combination (middle and lower). Macrophages only decreased in the vaccine and combination group.

As can be taken from Fig. 5c, effects on common signaling pathways in the combination group correlate more with the vaccine therapy than with *α*-PD-L1 treatment. In detail, the myeloid compartment, *TGF*-beta and *Wnt* signaling pathways were downregulated in all treatment groups in comparison with the control. Additionally in the combination group, genes related to angiogenesis and PI3K/Akt pathway were downregulated. For the latter, *LAMA1* and *Comp* were downregulated, whereas the phosphatase *PTEN*, a well-established tumor suppressor was upregulated (Fig. 5d). Genes for costimulatory signaling and lymphoid compartment were higher in tumors of mice receiving *α*-PD-L1 and the combination, whereas no changes were seen in the vaccine group. Furthermore, the JAK/STAT signaling was activated by the combination (Fig. 5d).

We summarize the detailed analysis of differential expression at the gene set level (supplementary Fig. 1). In the combination, genes belonging to the interferon signaling (such as *H2-Q1/H2-Q2, Ifi203,* and *Vcam1*) and cytotoxicity (*Gzma* and *Tnfsf10*) were upregulated, whereas all genes of the myeloid compartment genes were downregulated (including *Ly6C1*, *Olr1,* and *Ccl20*).

### Combination therapy alters the tumor microenvironment

**Fig. 6:**
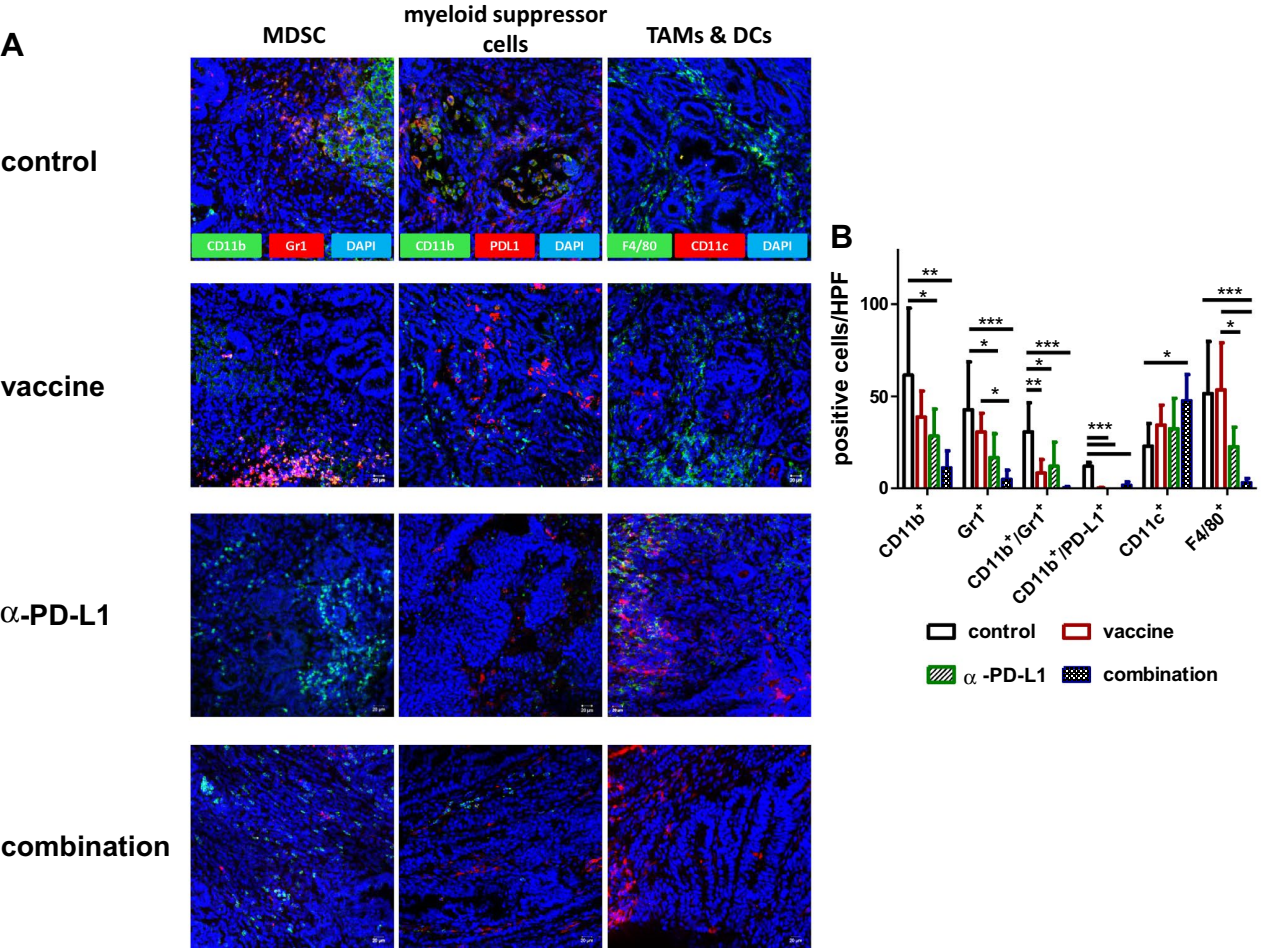

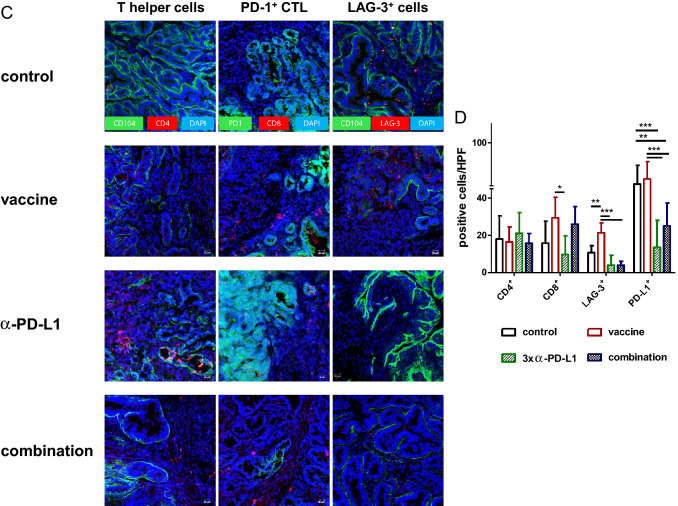
Immunofluorescence **a, c** Residual Mlh1^−/−^ GIT were resected after therapeutic vaccination and cryostat sections of 4 µm prepared. Tumor microenvironment was studied upon staining with specific mAbs, followed by nuclear staining with DAPI. Pictures were taken on a laser scanning microscope (Zeiss) using 20× objectives. **b, d** Quantification of infiltrating immune cells. At least three pictures were taken from each slide and numbers of infiltrating cells counted. Data are given as infiltrates/HPF. Mean + SD, *n* ≥ 3 samples/group; *p < 0.05, **p<0.01; ***p<0.001 one-way ANOVA (Bonferroni’s multiple comparison test)

While above findings already showed changes between individual treatment groups, we additionally studied the tumor microenvironment by immunofluorescence (Fig. 6).

MDSC and F4/80^+^ tumor-associated macrophages (TAM) were detectable in control tumors, indicative a suppressive microenvironment. While MDSC were effectively eliminated upon therapy, irrespective of the applied treatment regimen, only the combination was able to impact on numbers of infiltrating TAM (Fig. 6a, b). Besides, CD11c^+^ cells increased in the combination. Numbers of CD4^+^ T-helper cells remained the same, while the CD8^+^ CTL increased. This resulted in a significant difference between the vaccine and the *α*-PD-L1 treatment (Fig. 6c, d). Vice versa, the amount of LAG-3^+^ T-cells significantly increased upon vaccine treatment but significantly decreased upon *α*-PD-L1 and the combination. These results were similar to the levels of PD-L1^+^ cells, which significantly decreased in these two groups (Fig. 6c, d). Though PD1 was highly abundant on tumor cells, we observed intratumoral differences, ranging from high to low PD1 expression within individual specimen (supplementary Fig. 2). This was, however, treatment-independent.

### Treatment-induced molecular changes in cMS

**Fig. 7:**
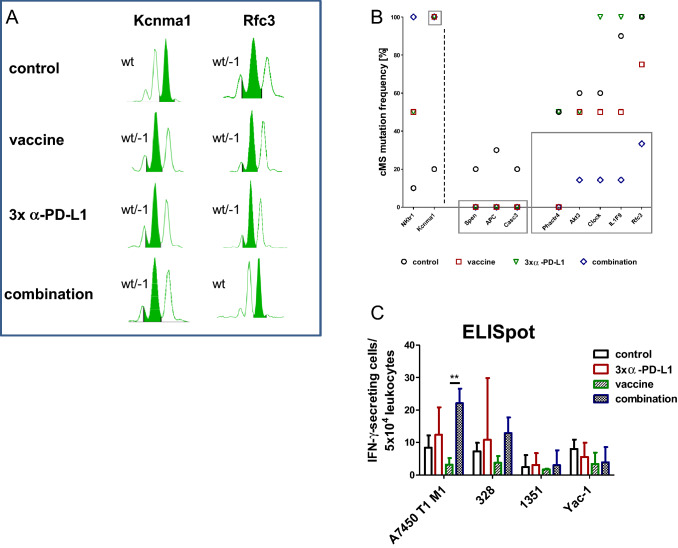
Fragment length analysis of cMS mutations in Mlh1^−/−^ target genes and IFN-*γ* ELISpot **a** Representative pattern of cMS markers. MSI is defined by mono- and/or bialellic band shifts characterized by deletions (indicated with minus symbol + number). The wild type peak is highlighted in green **b** Quantitative cMS analysis using a panel of predefined Mlh1^−/−^ target genes. Represented is the mutation frequency of selected target genes in mice from the control (*n* = 10), vaccine (*n* = 4), *α*-PD-L1 (*n *= 4), and combination 2 (*n* = 7). Note the differences in mutational frequency between control and treated mice indicating loss of single cell clones (= no mutation detected, such as *Spen, Apc, Casc3*) mainly in the combination 2 group (gray frame) **c** Reactivity of splenocytes against target cells (Mlh1^−/−^ 7450 T1 M1, Mlh1^−/−^ 328, Mlh1^−/−^ 1351, and YAC-1) was examined after overnight co-incubation. Lymphocytes were isolated from mice of the following groups: control (*n* = 3), vaccine (*n* = 5), *α*-PD-L1 (*n *= 4) and combination 2 (*n* = 4). Highest reactivity was seen in the combination treatment. Given is the mean ± SD, **p*<*0.01 one-way ANOVA (Bonferroni’s multiple comparison test)

Residual tumors of the different treatment groups were scanned for typical gene mutations at cMS (Fig. 7a, b). Depending on the treatment, tumors harbored different mutation frequencies in cMS. *NKtr1* and *Kcnma1* (left of the dotted line) had the lowest mutation rates for the control, whereas the treatments resulted in high mutation rates. Noticeably, GIT from all three treatment groups showed no mutation in *Spen*, *Apc* and *Casc3* (highlighted with the gray box in the middle), while a mutation rate of 20–30% was evidently in control tumors. Residual tumors from the combination harbored the lowest mutation frequencies in *Akt3*, *Clock*, *Il1F9* and *Rfc3* (highlighted with the right gray box), especially compared with *α*-PD-L1 treatment (= 100% mutation frequency).

### ELISpot analysis reveals increased immune activation upon combination treatment

To asses immune activation, IFN-*γ* secretion by T-lymphocytes was detected by ELISpot-assays after coincubation of splenocytes from treated and control mice with different cancer cell lines (A7450 T1 M1, 328, 1351, and Yac-1) (Fig. 7c). Splenocytes of mice from the combination group responded with significantly higher IFN-*γ* secretion than those treated with *α*-PD-L1. NK cell reactivity was excluded by lacking IFN-*γ* secretion against Yac-1 cells. Notably, IFN-*γ* secretion levels against 1351 MLH1^−/−^ lymphoma cells were the lowest irrespective of the treatment.

## Discussion

In this study, we describe a strategy to combine active tumor vaccination with an ICI in a clinically relevant dMMR mouse model [32]. DMMR is associated with high tumor mutational burden [33–35] and thus harbors a tentatively high likelihood of being susceptible to immunotherapy.

Using a murine *α*-PD-L1 antibody, monotherapy itself marginally improved outcome after single application. By increasing the number of injections, overall survival of Mlh1^−/−^ mice extended to a degree comparable to the vaccine monotherapy. The latter was prepared from a whole tumor lysate with proven antitumor activity from previous studies [27, 36]. Hence, both treatments prolonged mice’ survival suffering from highly aggressive Mlh1^−/−^-driven GIT. Given the fact that Mlh1^−/−^ tumors, despite their high TMB, do not have a high IFN*γ* signature and are not targetable by ICI per se, the improved outcome after *α*-PD-L1 monotherapy is intriguing. It is therefore unlikely that mice’ outcome after targeting the PD-L1 axis is better if *α*-PD-L1 antibodies are applied more often or over a longer time. Rather targeting both MHC-I and II restricted tumor epitopes—with whole tumor lysates—in combination with PD-L1 blockade seems necessary to affect growth of poorly immunogenic and thus ICI refractory, immunologically cold/warm tumors, as recently shown for triple-negative breast cancer [37]. So far, we can only speculate on the survival benefit of mice treated with the *α*-PD-L1 antibody in monotherapy. In a very recent study on dMMR gastric cancer, CD68^+^CD163^−^ M1-like macrophages were identified as prerequisites for efficient PD-L1/PD-1 blockade because of specific chemokine receptor expression likely activating CTL [38]. The *α*-PD-L1 antibody itself may have also induced immune-independent apoptosis and autophagy in Mlh1^−/−^ cells. In addition to our RNA expression data, showing signaling pathway alteration, increased release of reactive oxygen species and cytochrome-c was found in atezolizumab-treated osteosarcoma cells, ultimately leading to mitochondrial-related apoptosis [39].

Another interesting finding of our study was the upregulation of angiogenesis pathways under *α*-PD-L1 monotherapy, adding further credence for combined checkpoint-angiogenesis inhibition, currently tested in clinical trials [40, 41].

However, dosing schedules and accurate timing of each combination partner remain undefined for combined vaccine and ICI strategies. Here, we performed alternating treatment starting with vaccine first. The rationale is based on our previous observations in which repetitive vaccine monotherapy provoked upregulation of immune-checkpoint-molecules on residual tumors [27]. To counteract therapy-induced upregulation, we here applied *α*-PD-L1 therapy during vaccination. This combined treatment yielded complete remission in 30% of mice, finally resulting in significantly improved overall survival. Although complete remission was not achieved in all mice, we would like to stress the point that tumor burden massively reduced in the combination likely because of inducing a T cell–inflamed tumor microenvironment. Other studies reported superior effects when checkpoint-inhibition was given after cessation of the vaccine [42]. Still, the significantly prolonged overall survival of MLH1^−/−^ mice achieved in this study argues in favor of concomitant application. By applying dual immune-checkpoint blockade (such as *α*- or *α*-LAG-3) one may expect even better and long-term tumor growth control.

Most previous trials focused on *α*-PD-1 antibodies to increase antitumoral effects of vaccine-induced immunity [43–45]. Rare preclinical data exist on vaccine-*α*-PD-L1 combinations. A recent study described prolonged survival and increased tumor cell apoptosis in a hepatocellular carcinoma model treated with a combined DC vaccine and *α*-PD-L1 inhibitor [46], supported by findings from Ji et al., reporting reactivation of neoantigen-specific CTL by combined *α*-PD-L1 peptide vaccination [47]. Likewise, Sun et al. found enhanced tumor-antigen-specific immunity upon combined vaccine-PD-L1-blockade [48]. By reversing the immunosuppressive status of the micromilieu, PD-L1 is indeed a promising target. Here, we also identified a shaped tumor microenvironment accompanied by peripheral immune activation. By performing a detailed and longitudinal analysis, we found decreased numbers of circulating MDSC and T cell exhaustion markers after combined treatment. Accompanying in-depth gene expression analysis of residual tumors identified increased numbers of total TIL, mainly being cytotoxic T and B cells. Vice versa, levels of exhausted CD8^+^ T cells, tumor-associated macrophages and neutrophils reduced in the combination group. Neutrophils are a group of tumor-associated cells which, in conjunction with MDSC, play a major role during cancer development and progression. Their specific location within the tumor (i.e., intra-, peritumoral or stromal) has prognostic relevance [49]. Abundance of tumor-associated neutrophils may even correlate with local TGFβ expression; in fact, TGFβ blocking improves outcome in preclinical cancer models [49]. In support of this, TGF-signaling was downregulated here upon combination and likely facilitated conquering primary resistance to checkpoint inhibition [50]. Though not analyzed in detail here, reduced TGFβ signaling may have also exerted a tumor-intrinsic effect finally blocking the EMT-like transition and preventing Mlh1^−/−^-driven tumor progression [50]. Additional common pathways with prognostic relevance that were altered by the vaccine-*α*-PD-L1 combination include PI3K/Akt and Wnt-signaling as well as genes responsible for angiogenesis, matrix remodeling and metastasis. By contrast, genes belonging to the JAK/STAT signaling were upregulated, indicative for enhanced immune-related crosstalk to eradicate Mlh1^−/−^ tumor cells via IFN-*γ* [51]. These cumulative data nicely explain the improved overall survival in mice treated with the combined vaccine-*α*-PD-L1 approach.

While most pronounced effects were in fact seen in the combination therapy and thus interpretable as synergistic, the monotherapy itself modulated the tumor microenvironment. Anti-PD-L1 treatment-induced genes relevant for autophagy and downregulated NF-κB-signaling, which is in line with data from a recent trial on triple-negative breast cancer cells, treated with Atezolizumab [52]. Upon vaccination, matrix remodeling/metastasis-related genes and genes of the Wnt-and TGF-signaling were downregulated and a direct indicator of successful reversal of intrinsic resistance. Indeed, tumor-intrinsic *β*-catenin activation prevents T cell priming and infiltration into the tumor microenvironment and results in resistance to anti-PD-L1/anti-CTLA-4 therapy [53]. Vice versa, Wnt-pathway suppression restores DC infiltration, a phenomenon seen here upon therapy characterized by elevated levels of tumor-infiltrating CD11c^+^ DC that confirm successful therapy-related downregulation of the Wnt-pathway.

Inter-individual differences throughout the treatment groups reflect the different overall survival times of mice. Here, short-term survivors had low TIL scores and vice versa. Teasing out what are the (patient-) individual baseline differences is the challenge for the next wave of pre- and clinical trials with immunotherapy to refine treatment on the long run.

Another interesting finding was the altered molecular profile in typical cMS marker upon treatment. One may speculate that treatment successfully eliminated single mutated clones, whereas other emerged under the immune-selective pressure. We identified somatic cMS mutations in *NKtr1* and *Kcnma1* in all treatment groups that were infrequent in control tumors. By contrast, somatic mutations in *Spen*, *Apc,* and *Casc3* were no longer detectable. Notably, residual tumors from the combination therapy harbored the lowest mutation frequencies in *Akt3*, *Clock*, *Il1F9,* and *Rfc3*, especially compared with *α*-PD-L1 treatment (= 100% mutation frequency).

Among others, question remains why some tumors regressed, while others finally progressed. Sustained tumor IFN signaling induces PD-L1 expression on tumor and immune cells and is considered a acquired resistance mechanism [54]. However, this only partially explains the different in vivo response. Reports from human dMMR CRC describe contradictory PD-L1 abundance on tumor-infiltrating lymphocytes or tumor cells [55, 56]. Its role to mediate immune escape is undebatable and results from a phase II study already confirmed antitumor activity of Avelumab with manageable toxicity in most, but clearly not all patients with previously treated dMMR mCRC and recurrent/persistent endometrial cancer [26, 57]. Heterogeneity among tumors, such as the varying TMB, different genomic variations (in cMS), Indoleamine 2,3-Dioxygenase 1-based immune escape, and the activated Wnt/*β*-catenin signaling may provide an explanation for the difference seen here. Understanding how Mlh1^−/−^ tumor and immune cells react to our treatments holds promise for novel immune-modulating strategies and will hopefully help to guide the way for clinical vaccine-based immune-checkpoint regimens.

## Conclusion

Tumor-lysate vaccination in combination with *α*-PD-L1 prolongs the lifetime of Mlh1 knock-out mice significantly and shows strong tumor growth inhibition via downregulation of PI3K/Akt/Wnt-and TGF-signaling. This combination regimen results in decreased levels of myeloid-derived suppressor cells (MDSC), splenic and intratumoral checkpoint-expressing T cells (PD-L1, LAG-3 and CTLA-4) and therefore positively modulates the tumor microenvironment. Combined vaccine-immune-checkpoint inhibition provides a safe approach especially for patients having a likelihood of being non-responsive toward immune-checkpoint monotherapy.

## Supplementary Information

Below is the link to the electronic supplementary material.

Supplementary file1 (PPTX 3359 kb)

## Data Availability

The datasets used and/or analyzed during the current study are available from the corresponding author on reasonable request.

## Abbreviations

cMS: Coding microsatellite
CTL: Cytotoxic T-lymphocytes
DC: Dendritic cells
dMMR: Mismatch repair deficiency
GIT: Gastrointestinal tumor
ICI: Immune-checkpoint inhibitors
MDSC: Myeloid-derived suppressor cells
TMB: Tumor mutational burden

## References

[1] Cousin S, Seneschal J, Italiano A (2018). Toxicity profiles of immunotherapy. Pharmacol Ther.

[2] Pistillo MP, Carosio R, Grillo F (2020). Phenotypic characterization of tumor CTLA-4 expression in melanoma tissues and its possible role in clinical response to Ipilimumab. Clin Immunol.

[3] Suzman DL, Agrawal S, Ning Y (2019). FDA approval summary: Atezolizumab or Pembrolizumab for the treatment of patients with advanced Urothelial carcinoma ineligible for cisplatin-containing chemotherapy. Oncologist.

[4] Crist M, Balar A (2017). Atezolizumab in invasive and metastatic urothelial carcinoma. Expert Rev Clin Pharmacol.

[5] Plimack ER, Bellmunt J, Gupta S (2017). Safety and activity of pembrolizumab in patients with locally advanced or metastatic urothelial cancer (KEYNOTE-012): a non-randomised, open-label, phase 1b study. Lancet Oncol.

[6] Leng C, Li Y, Qin J (2016). Relationship between expression of PD-L1 and PD-L2 on esophageal squamous cell carcinoma and the antitumor effects of CD8+ T cells. Oncol Rep.

[7] Kalim M, Iqbal Khan MS, Zhan J (2020). Programmed cell death ligand-1: a dynamic immune checkpoint in cancer therapy. Chem Biol Drug Des.

[8] Liu J, Zhang S, Hu Y (2016). Targeting PD-1 and Tim-3 pathways to reverse CD8 T-cell exhaustion and enhance ex vivo T-cell responses to autologous dendritic/tumor vaccines. J Immunother.

[9] Siegel RL, Torre LA, Soerjomataram I (2019). Global patterns and trends in colorectal cancer incidence in young adults. Gut.

[10] Evrard C, Tachon G, Randrian V (2019). Microsatellite Instability: diagnosis, heterogeneity, discordance, and clinical impact in colorectal cancer. Cancers (Basel).

[11] Ait Ouakrim D, Dashti SG, Chau R (2015). Aspirin, Ibuprofen, and the Risk of Colorectal Cancer in Lynch Syndrome. J Natl Cancer Inst.

[12] Seth S, Ager A, Arends MJ, Frayling IM (2018). Lynch syndrome – cancer pathways, heterogeneity and immune escape. J Pathol.

[13] Bodo S, Colas C, Buhard O (2015). Diagnosis of constitutional mismatch repair-deficiency syndrome based on microsatellite instability and lymphocyte tolerance to methylating agents. Gastroenterology.

[14] Wimmer K, Kratz CP (2010). Constitutional mismatch repair-deficiency syndrome. Haematologica.

[15] Luchini C, Bibeau F, Ligtenberg MJL (2019). ESMO recommendations on microsatellite instability testing for immunotherapy in cancer, and its relationship with PD-1/PD-L1 expression and tumour mutational burden: a systematic review-based approach. Ann Oncol.

[16] Le DT, Durham JN, Smith KN (2017). Mismatch repair deficiency predicts response of solid tumors to PD-1 blockade. Science.

[17] Campbell BB, Light N, Fabrizio D (2017). Comprehensive analysis of hypermutation in human cancer. Cell.

[18] Lemery S, Keegan P, Pazdur R (2017). First FDA approval agnostic of cancer site—when a biomarker defines the indication. N Engl J Med.

[19] Loupakis F, Depetris I, Biason P (2020). Prediction of benefit from checkpoint inhibitors in mismatch repair deficient metastatic colorectal cancer: role of tumor infiltrating lymphocytes. Oncologist.

[20] Ariyan CE, Brady MS, Siegelbaum RH (2018). Robust antitumor responses result from local chemotherapy and CTLA-4 blockade. Cancer Immunol Res.

[21] Bozorgmehr F, Hommertgen A, Krisam J (2019). Fostering efficacy of anti-PD-1-treatment: nivolumab plus radiotherapy in advanced non-small cell lung cancer - study protocol of the FORCE trial. BMC Cancer.

[22] Sullivan RJ, Hamid O, Gonzalez R (2019). Atezolizumab plus cobimetinib and vemurafenib in BRAF-mutated melanoma patients. Nat Med.

[23] Sahin IH, Akce M, Alese O (2019). Immune checkpoint inhibitors for the treatment of MSI-H/MMR-D colorectal cancer and a perspective on resistance mechanisms. Br J Cancer.

[24] Kong P, Wang J, Song Z (2019). Circulating lymphocytes, PD-L1 expression on tumor-infiltrating lymphocytes, and survival of colorectal cancer patients with different mismatch repair gene status. J Cancer.

[25] Le DT, Uram JN, Wang H (2015). PD-1 blockade in tumors with mismatch-repair deficiency. N Engl J Med.

[26] Kim JH, Kim SY, Baek JY (2020). A phase II study of avelumab monotherapy in patients with mismatch repair-deficient/microsatellite instability-high or *pole*-mutated metastatic or unresectable colorectal cancer. Cancer Res Treat.

[27] Maletzki C, Gladbach YS, Hamed M (2018). Cellular vaccination of MLH1^−/−^ mice–an immunotherapeutic proof of concept study. Oncoimmunology.

[28] Maletzki C, Wiegele L, Nassar I (2019). Chemo-immunotherapy improves long-term survival in a preclinical model of MMR-D-related cancer. J Immunother Cancer.

[29] Maletzki C, Gladbach YS, Hamed M (2018). Cellular vaccination of MLH1^−/−^mice–an immunotherapeutic proof of concept study. Oncoimmunology.

[30] Maletzki C, Beyrich F, Hühns M (2016). The mutational profile and infiltration pattern of murine MLH1^-/-^ tumors: concurrences, disparities and cell line establishment for functional analysis. Oncotarget.

[31] Maletzki C, Huehns M, Bauer I (2017). Frameshift mutational target gene analysis identifies similarities and differences in constitutional mismatch repair-deficiency and lynch syndrome. Mol Carcinog.

[32] Edelmann W, Yang K, Kuraguchi M (1999). Tumorigenesis in Mlh1 and Mlh1 / Apc1638N mutant mice. Cancer Res.

[33] Chae YK, Anker JF, Bais P, Namburi S (2018). Mutations in DNA repair genes are associated with increased neo-antigen load and activated T cell infiltration in lung adenocarcinoma. Oncotarget.

[34] Chang L, Chang M, Chang HM, Chang F (2018). Microsatellite instability: a predictive biomarker for cancer immunotherapy. Appl Immunohistochem Mol Morphol.

[35] Salem ME, Puccini A, Grothey A (2018). Landscape of tumor mutation load, mismatch repair deficiency, and PD-L1 expression in a large patient cohort of gastrointestinal cancers. Mol Cancer Res.

[36] Maletzki C, Wiegele L, Nassar I (2019). Chemo-immunotherapy improves long- term survival in a preclinical model of MMR-D-related cancer. J Immunother Cancer.

[37] Feola S, Capasso C, Fusciello M (2018). Oncolytic vaccines increase the response to PD-L1 blockade in immunogenic and poorly immunogenic tumors. Oncoimmunology.

[38] Zhao R, Wan Q, Wang Y (2021). M1-like TAMs are required for the efficacy of PD-L1/PD-1 blockades in gastric cancer. Oncoimmunology.

[39] Liu Z, Wang H, Hu C (2021). Targeting autophagy enhances atezolizumab-induced mitochondria-related apoptosis in osteosarcoma. Cell Death Dis.

[40] Lee MS, Ryoo BY, Hsu CH (2020). Atezolizumab with or without bevacizumab in unresectable hepatocellular carcinoma (GO30140): an open-label, multicentre, phase 1b study. Lancet Oncol.

[41] McDermott DF, Huseni MA, Atkins MB (2018). Clinical activity and molecular correlates of response to atezolizumab alone or in combination with bevacizumab versus sunitinib in renal cell carcinoma. Nat Med.

[42] Kodumudi KN, Ramamoorthi G, Snyder C (2019). Sequential Anti-PD1 therapy following dendritic cell vaccination improves survival in a HER2 mammary carcinoma model and identifies a critical role for CD4 T cells in mediating the response. Front Immunol.

[43] Yoo SY, Badrinath N, Jeong S-N (2020). Overcoming tumor resistance to oncolyticvaccinia virus with Anti-PD-1-based combination therapy by inducing antitumor immunity in the tumor microenvironment. Vaccines.

[44] Kadam P, Sharma S (2020). Pd-1 immune checkpoint blockade promotes therapeutic cancer vaccine to eradicate lung cancer. Vaccines.

[45] Xu G, Feng D, Yao Y (2020). Listeria-based hepatocellular carcinoma vaccine facilitates anti-PD-1 therapy by regulating macrophage polarization. Oncogene.

[46] Teng CF, Wang T, Wu TH (2020). Combination therapy with dendritic cell vaccine and programmed death ligand 1 immune checkpoint inhibitor for hepatocellular carcinoma in an orthotopic mouse model. Ther Adv Med Oncol.

[47] Ji S, Lee J, Lee ES (2021). B16 melanoma control by anti-PD-L1 requires CD8+ T cells and NK cells: application of anti-PD-L1 Abs and Trp2 peptide vaccines. Hum Vaccines Immunother.

[48] Sun NY, Chen YL, Wu WY (2019). Blockade of PD-L1 enhances cancer immunotherapy by regulating dendritic cell maturation and macrophage polarization. Cancers (Basel).

[49] Shaul ME, Fridlender ZG (2019). Tumour-associated neutrophils in patients with cancer. Nat Rev Clin Oncol.

[50] Canè S, Van Snick J, Uyttenhove C (2021). TGFβ1 neutralization displays therapeutic efficacy through both an immunomodulatory and a non-immune tumor-intrinsic mechanism. J Immunother Cancer.

[51] Rieth J, Subramanian S (2018). Mechanisms of intrinsic tumor resistance to immunotherapy. Int J Mol Sci.

[52] Saleh R, Taha RZ, Nair VS (2019). PD-L1 blockade by atezolizumab downregulates signaling pathways associated with tumor growth, metastasis, and hypoxia in human triple negative breast cancer. Cancers (Basel).

[53] Spranger S, Bao R, Gajewski TF (2015). Melanoma-intrinsic *β*-catenin signalling prevents anti-tumour immunity. Nature.

[54] Benci JL, Xu B, Qiu Y (2016). Tumor interferon signaling regulates a multigenic resistance program to immune checkpoint blockade. Cell.

[55] Ho HL, Chou TY, Yang SH (2019). PD-L1 is a double-edged sword in colorectal cancer: the prognostic value of PD-L1 depends on the cell type expressing PD-L1. J Cancer Res Clin Oncol.

[56] Korehisa S, Oki E, Iimori M (2018). Clinical significance of programmed cell death-ligand 1 expression and the immune microenvironment at the invasive front of colorectal cancers with high microsatellite instability. Int J Cancer.

[57] Konstantinopoulos PA, Luo W, Liu JF (2019). Phase II study of avelumab in patients with mismatch repair deficient and mismatch repair proficient recurrent/persistent endometrial cancer. J Clin Oncol.

