# Supplementary figures and images for: Combined vaccine-immune-checkpoint inhibition constitutes a promising strategy for treatment of dMMR tumors
Source: Cancer Immunol Immunother. 2021 Apr 18;70(12):3405–19. doi: 10.1007/s00262-021-02933-4 (PMC8571220; doi:10.1007/s00262-021-02933-4)

## Slide 1
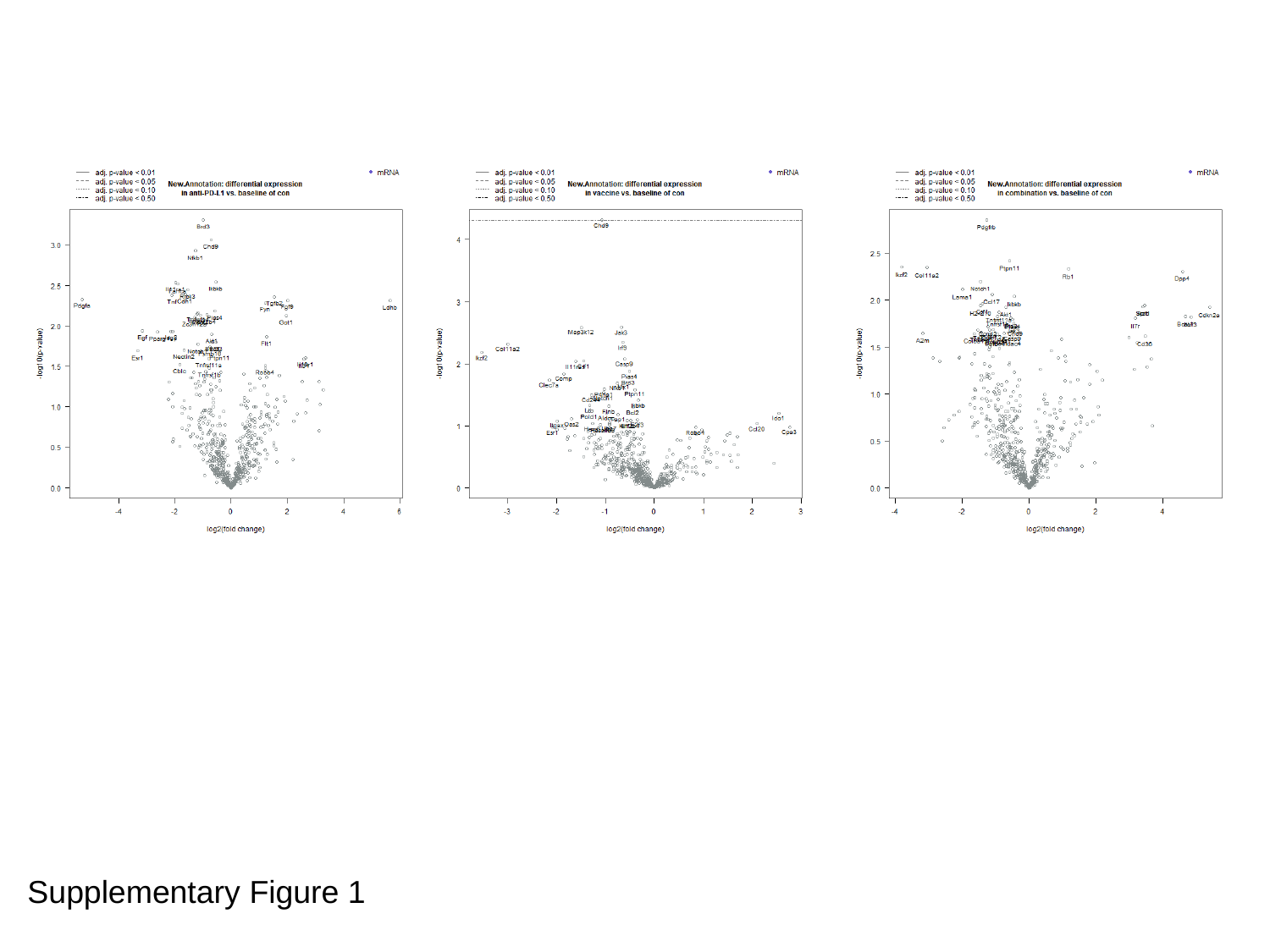

Supplementary Figure 1

## Slide 2
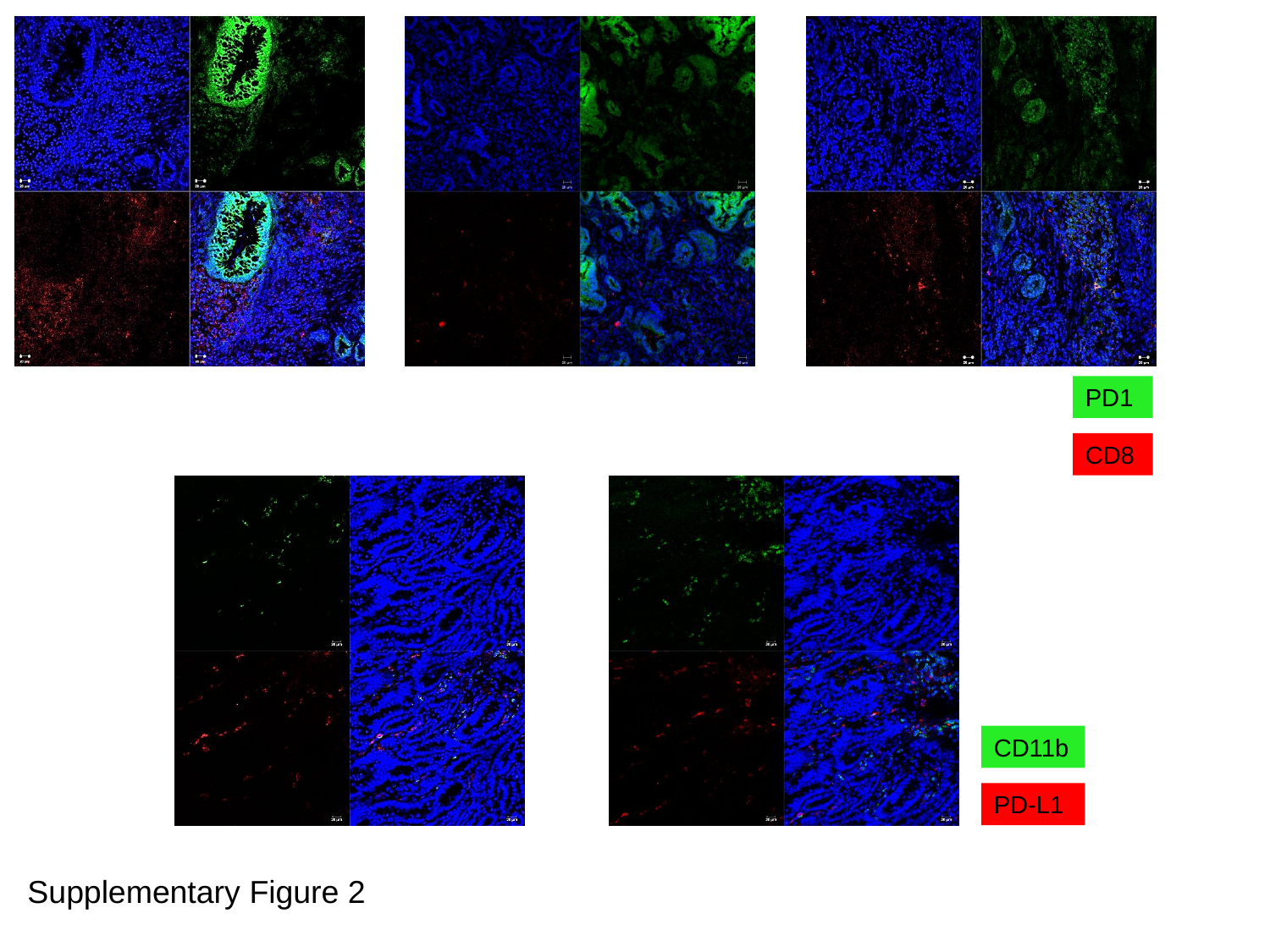

PD1
CD8
CD11b
PD-L1
Supplementary Figure 2

Supplement: Supplementary file 1 — Supplementary file1 (PPTX 3359 kb) [file 262_2021_2933_MOESM1_ESM.pptx]
